# Anterolateral Corrective Lumbar Corpectomy and Interbody Fusion by Using Extended Screw Fixation without Posterior Instrumentation for Posttraumatic Kyphosis

**DOI:** 10.1155/2013/614757

**Published:** 2013-07-18

**Authors:** Atsuro Yamazaki, Sumihisa Orita, Takeshi Sainoh, Kazuyo Yamauchi, Miyako Suzuki, Yoshihiro Sakuma, Go Kubota, Yasuhiro Oikawa, Kazuhide Inage, Yukio Nakata, Gen Inoue, Yasuchika Aoki, Tomoaki Toyone, Junichi Nakamura, Masayuki Miyagi, Kazuhisa Takahashi, Seiji Ohtori

**Affiliations:** Department of Orthopaedic Surgery, Graduate School of Medicine, Chiba University, Chiba 260-8670, Japan

## Abstract

A 26-year-old paraplegic schizophrenic Japanese woman suffered from severe kyphosis and back pain derived from lumbar burst fractures caused by jumping. She had already undergone resection of the L1 and L2 spinous processes for sharp angular kyphosis, but she still had severe kyphosis and back pain at the L1 and L2. Radiographical examination revealed fused anterior columns at L1 and L2 with severe local kyphosis and a significantly decreased percutaneous distance in the back. The patient underwent anterior instrumented bony resection including an L2 vertebral osteotomy: bilateral L2-L3 facetectomy and partial posterior osteotomy of the L2 vertebrae via a posterior approach followed by an anterior corpectomy of the L2 vertebrae and insertion of a cylindrical cage. No posterior instrumentation was used owing to the presence of atrophied paraspinal soft tissues. Lumbar interbody fusion was performed with vertebral body screws extending from T12 to L4 and corresponding anterior distension and posterior compression. The procedure corrected the kyphosis by 15° and enhanced local stability. Postsurgical visual analogue scale improved from 9.0 to 2.0 and Oswestry Disability Index from 40 to 17.8, respectively. In conclusion, we have demonstrated that anterolateral interbody fusion using extended fixation can compensate for posterior corrective surgery.

## 1. Introduction

Posttraumatic kyphosis is a common sequela of inadequately managed thoracolumbar fractures leading to severe back pain at the apex of the kyphosis and impairs physical activity and quality of life [1, 2]. Corrective surgical procedures using rigid posterior instrumentation should be performed for these patients, however sometimes inappropriate for some patients.

## 2. Case Presentation

The case was a 26-year-old paraplegic woman complaining of severe kyphotic deformity and back pain derived from lumbar burst fractures. She had been diagnosed as a schizophrenic and had jumped from a height of about 65 feet resulting in L1 and L2 vertebral burst fractures with paraplegia (Frankel A). Because of her unstable medical condition for weeks, she was treated nonoperatively which led to bony fusion in the local kyphotic alignment. At the following spine center she complained of back pain around the L1 and L2 spinal processes (the apex of the kyphosis). The thin, distracted skin around the processes was considered to be the pain origin, so she underwent resection of the processes, but this treatment failed. She was referred to our hospital for possible improvement of her pain and kyphosis. 

**Figure 1:**
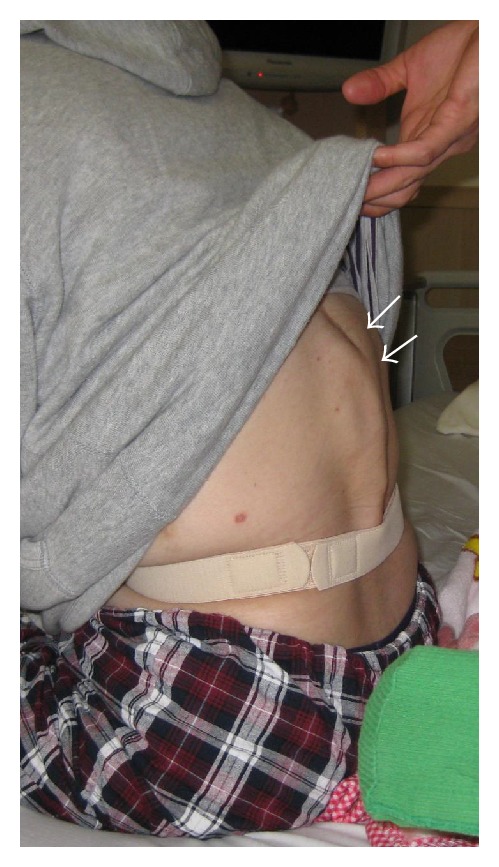
A 26-year-old patient presented with severe kyphosis and back pain caused by lumbar burst fractures with fused L1 and L2 anterior columns (arrows).

**Figure 2:**
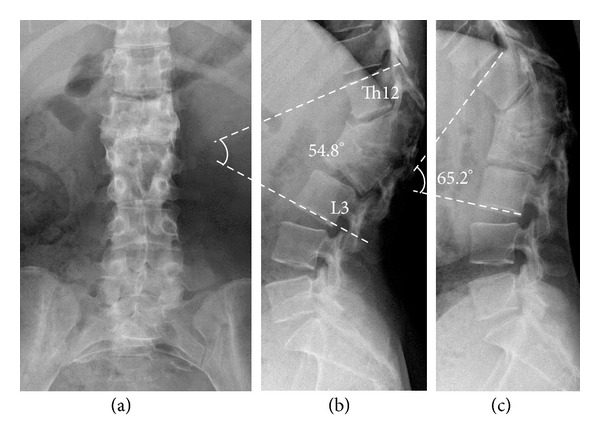
Presurgical plain radiographs: (a) AP view showing the bony fusion of the L1 and L2 vertebrae. (b) Lateral neutral view showing 54.8° of local kyphosis from T12 to L3. (c) Lateral flexion view showing worsening of the kyphosis to 65.2°.

**Figure 3:**
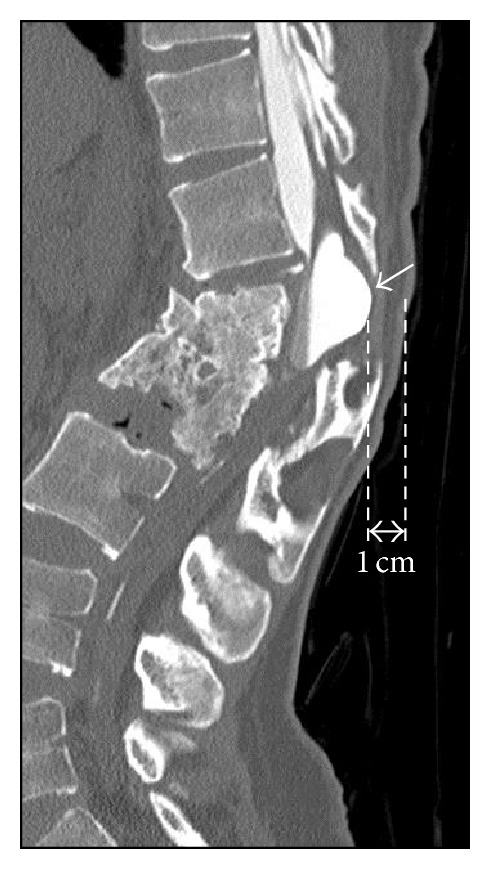
Computed tomography myelography (CTM) revealed a pouch-like total blockage at the L1 level (short arrow) and a 1 cm distance between the body surface and the dura mater with atrophied back muscles.

The patient showed complete paraplegia with subumbilical anesthesia and local kyphosis with back pain at the levels of L1 and L2, which worsened in intensity and frequency whilst sitting. She was unable to sit down for long periods or to lie on her back owing to the protrusion of the edges of the residual spinous processes (Figure 1). Her visual analogue scale (VAS) score was 8.5 for back pain and 9.0 for percutaneous pain around the apex of the kyphosis, and Oswestry Disability Index (ODI) was scored as 40. Radiography showed severe acute kyphosis due to the L1 and L2 vertebral fractures with anterior bony fusion (Figure 2). The local kyphosis was 54.8° from T12 to L3 in the upright position and worsened to 65.2° during flexion. Myelography and computed tomography myelography (CTM) revealed a pouch-like total blockage at the L1 level and a 1 cm distance between the body surface and the dura mater with atrophied back muscles (Figure 3). 

**Figure 4:**
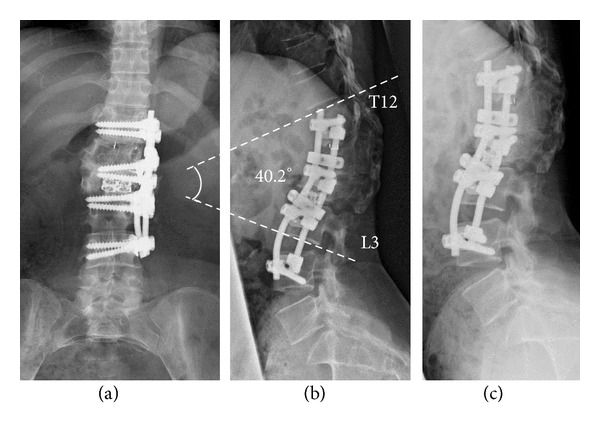
Postsurgical plain radiographs: (a) AP view showing the anterolateral fusion from T12 to L4 with interbody fusion between L2 and L3. (b) Lateral neutral view showing improved alignment measuring 40.2° from T12 to L3 and approximate 15° correction. (c) Lateral flexion view showing the stabilized lumbar spine.

**Figure 5:**
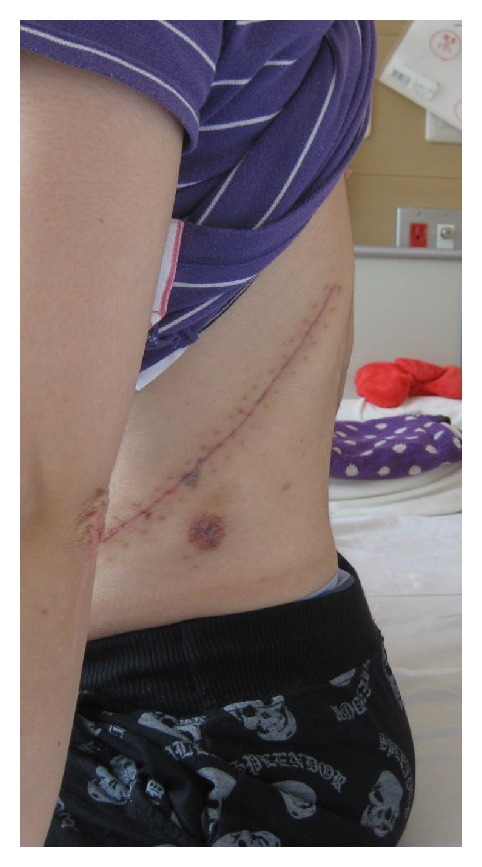
Presurgical severe kyphosis was dramatically improved with significant pain relief.

The patient underwent a posterior bony resection to reduce kyphotic prominence including an L2 dorsal vertebral osteotomy. First, a bilateral L2-L3 facetectomy and partial posterior osteotomy of the L2 vertebrae were performed via the posterior approach. Posterior instrumentation was not used owing to the presence of atrophied paraspinal soft tissues, which limited the available space. Thereafter, a left side extrapleural-retroperitoneal approach was taken followed by anterior corpectomy of the L2 vertebrae and the insertion of a cylindrical cage. Lumbar interbody fusion was performed using the anterolateral approach with the vertebral body screw fixation extending from T12 to L4 with two rods, providing corresponding anterior distension and posterior compression, respectively (Figure 4). CD Horizon system (Medtronic, Memphis, TN, USA) was used for instrumentation. The procedure corrected the kyphosis by approximately 15° and provided enhanced local stability (Figures 4 and 5). The postsurgical VAS scores for both kinds of pain showed drastic improvements to around 2.0. ODI showed improvement to 17.8. The patient showed no complications such as recurrence of pain, implant failure, and increased malalignment during the follow-up period of 2 years.

## 3. Discussion 

Patients with kyphotic deformities >30° have been reported to be at increased risk for chronic back pain [3–5], which was confirmed in the present case. Generally, posttraumatic kyphosis requires correcting surgery such as pedicle subtraction osteotomy (PSO) or vertebral column resection [6]. However, rigid and posterior instrumentation techniques were not available for our patient owing to the atrophied paraspinal soft tissues, which increased the risk of soft tissue ischemia, wound dysraphia, and local infection. In addition, her presurgical radiographic findings suggested severe synechia and possible difficulty around the dura mater. We therefore performed extended corrective lumbar interbody fusion via an anterolateral approach without posterior instrumentation. The procedure corrected the sagittal alignment, provided sufficient stability, and decreased lower back pain. 

PSO is reported to be superior to anterior corpectomy and plating for correcting posttraumatic kyphosis [7], which could imply the possible insufficient stability in the present case. However, an anterior plate or posterior instrumentation following anterior lumbar interbody fusion has been shown as biomechanically stable regardless of the plate location [8]. Also corrective PSO can give the ability of soft tissue coverage of instrumentation by increasing lordosis, which is not acceptable in the present case for the severely damaged soft tissue at the posterior kyphotic site. Another study reported that extended anterolateral fixation is biomechanically comparable to circumferential fusion in the treatment of unstable thoracolumbar burst fractures with posterior column involvement [9]. These reports support our validity of the procedure with extended fusion ranging from T12 to L4.

## 4. Conclusion

We performed an anterolateral corrective corpectomy and interbody fusion using extended fusion from T12 to L4 on a posttraumatic local kyphosis without any posterior instrumentation that acquired enough correction, stability, and pain relief.
